# LncRNAs as an intermediate in HPV16 promoting myeloid-derived suppressor cell recruitment of head and neck squamous cell carcinoma

**DOI:** 10.18632/oncotarget.14939

**Published:** 2017-02-01

**Authors:** Xiangrui Ma, Surui Sheng, Jingbiao Wu, Yaping Jiang, Xiaolei Gao, Xiao Cen, Jiashun Wu, Shasha Wang, Yajie Tang, Yaling Tang, Xinhua Liang

**Affiliations:** ^1^ State Key Laboratory of Oral Diseases, West China Hospital of Stomatology (Sichuan University), Chengdu, Sichuan 610041, China; ^2^ Department of Oral and Maxillofacial Surgery, Affiliated Hospital of Binzhou Medical College, Binzhou, Shandong 256600, China; ^3^ Department of Implant, The Affiliated Hospital of Qingdao University, Qingdao, Shandong 266000, China; ^4^ Key Laboratory of Fermentation Engineering (Ministry of Education), Hubei University of Technology, Wuhan, Hubei 430068, China; ^5^ Department of Oral Pathology, West China Hospital of Stomatology (Sichuan University), Chengdu, Sichuan 610041, China; ^6^ Department of Oral and Maxillofacial Surgery, West China Hospital of Stomatology (Sichuan University), Chengdu, Sichuan 610041, China

**Keywords:** head and neck squamous cell carcinomas (HNSCC), human papillomavirus (HPV), the non-coding RNA (lncRNA), myeloid-derived suppressor cells (MDSCs)

## Abstract

The emerging evidence showed that long noncoding RNAs (lncRNAs) are involved in cell growth and apoptosis as well as cancer progression and metastasis of malignant tumor, however, limited data are available on the role of lncRNAs in human papillomavirus (HPV)-associated Head and neck squamous cell carcinomas (HNSCC). Here, we demonstrated that 23.98% of 196 HNSCC cases in Southwest China could be classified as HPV16 infection. The number of MDSCs in HPV-positive HNSCC was significantly higher than normal control, indicating that HPV infection may promote MDSCs aggregation. Then, we applied an array-based approach to monitor the lncRNA expression between HPV-positive HNSCC, HPV-negative HNSCC and normal oral mucous, and obtained 132 different lncRNAs in different HPV infected states of HNSCC. HOTAIR, PROM1, CCAT1, and MUC19 mRNA levels, determined by qRT-PCR were inversely correlated with MDSCs collection of HPV-associated HNSCC in 2 independent patient cohorts. The results may provide a rationale for the further evaluation of lncRNAs as a molecular target to elucidate the molecular mechanism of HPV promoting MDSCs collection of HNSCC.

## INTRODUCTION

Head and neck squamous cell carcinoma (HNSCC) is considered as one of the most prevalent and lethal cancers worldwide [1, 2]. Tobacco and alcohol are the strongest etiologic factors [3], and high-risk human papillomavirus (HPV), commonly HPV16, is an emerging etiologic factor in HNSCC [4–8]. Clinical reports provide clear evidence of improved outcome in patients with HPV-positive HNSCC relative to HPV-negative HNSCC [9, 10]. However, the underlying molecular mechanisms responsible for differences in the clinical behavior of patients with HPV-positive and HPV-negative HNSCC remains rather limited [11].

Long non-coding RNAs (lncRNAs), with length longer than 200 nucleotides, work by modifying chromatin and adjusting the networks of genetic and signal pathways in the pathogenesis of cancer [12]. The aberrantly expressed lncRNAs were closely associated with the progression and metastasis of colorectal cancer [13], prostate cancer [14], hepatocellular carcinoma [15], thyroid carcinoma [16], and tongue squamous cell carcinoma [17]. Recently, lncRNA is emerging as a biomarker with diagnosis value in the personalized treatment of the inflammation-related alterations, and has been regarded a link between inflammation and cancer. LncRNA NKILA can directly interact with functional domains of signaling proteins as a class of NF-κB modulators to inhibit cancer metastasis [18]. Arid2-IR, a Smad3-associated lncRNA, functions to promote NF-κB-dependent renal inflammation and the inhibition of Arid2-IR might represent a novel and specific therapy of renal inflammatory disease [19]. These demonstrated that lncRNAs can recruit inflammation. On the other hand, HPV 16 E6 upregulated lncRNA DNMT1 expression in HPV 16 positive cervical cancer cells SiHa and CaSki, indicating that lncRNA may play an important role in HPV 16-positive carcinoma [20]. Thus, we hypothesized that lncRNAs involved in transcriptional and posttranscriptional regulation of HPV infection-triggered inflammation alteration in HNSCC.

A better understanding of molecular principles will result in innovative concepts for precision medicine of both HPV-positive and HPV-negative HNSCC, based on the etiology and biology of these tumors. Consequently, we focused our attention on HPV dependent alterations of lncRNAs [21]. Here, in 180 fresh tumor tissues and 196 peripheral blood samples of HNSCC we examined the positive rate of HPV 16 DNA, and the number of myeloid-derived suppressor cells (MDSCs), a heterogeneous population of cells that consists of myeloid progenitor cells and immature myeloid cells [22]. Furthermore, we applied an array-based approach to monitor lncRNA expression between HPV-negative HNSCC, HPV-positive HNSCC and normal oral mucous, and obtained different lncRNAs confirmed by qRT-PCR. The results may uncover the role of lncRNAs as molecular targets in HPV-positive HNSCC.

## RESULTS

### HPV16 DNA expression in peripheral blood and fresh tumor tissue samples of HNSCC in Southwest China

A total of 288 patients with HNSCC were confirmed as eligible for the study. Among these, 62 patients rejected to take part in this research and 30 did not finish their questionnaires, leaving 196 enrolled case subjects and a case participation rate of 68.06%. The samples from 180 fresh primary tumor tissues, 196 peripheral blood samples and 196 paraffin sections from the same patient were successfully obtained (Supplementary Figure 1). To our knowledge, this is the maximum number of human fresh tumor tissue and blood samples of HNSCC patients to examine HPV 16 expression to date. In addition, to investigate the development of oral mucous lesion to HNSCC, we collected 30 precancerous lesions and 30 normal oral mucous of healthy persons at the same time.

**Figure 1:**
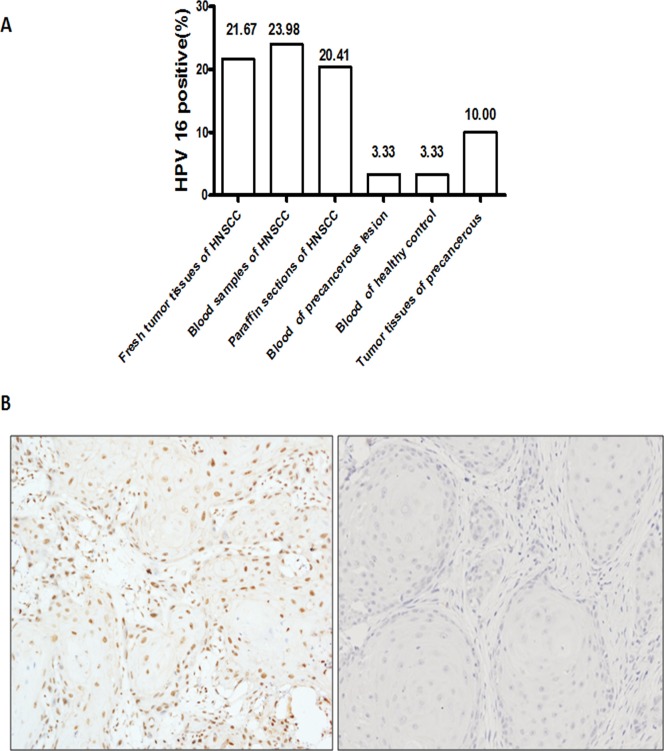
HPV 16 infection status of HNSCC patients **A**. The rate of HPV in HNSCC patients by different samples and detections. The level of HPV 16 infection in HNSCC group was significantly higher than the precarcinoma and normal oral mucous group (*P*<0.001). **B**. The representative figures of P16 immunostaining in HNSCC patients were shown. Right was P16 in strong positive staining. Left was P16 in negative staining.

180 fresh tumor tissues and 196 blood samples with available tumor DNA were successfully assayed by HPV 16 or HPV 18 viral DNA PCR, and 196 paraffin sections were examined by P16 immunostaining and HPV 16 DNA PCR. The result showed that the positive expression of HPV16 and 18 in peripheral blood was shown in 23.98% (47/196) and 6.12% (12/196) of HNSCC, respectively (Figure 1A). The positive of HPV16 and 18 in fresh tumor tissue was shown in 21.67% (39/180) and 5.00% (9/180) of HNSCC, respectively. Due to the low level of HPV 18 in HNSCC, we have not further examined the HPV 18 in the following study. In paraffin sections, the overall prevalence of HPV16 DNA by PCR was 20.41% (40/196), and the prevalence of P16 immunohistochemistry was 26.53% (52/196). The representative figures of P16 immunohistochemistry staining was shown in Figure 1B. These data indicated that HPV 16 infection prevalence of different samples and detection methods was coincident in 196 HNSCC patients. In addition, there were 2 cases with HPV 16 infection in the peripheral blood sample of precancerous lesion and normal oral mucous, and 3 cases in tumor tissues of precancerous lesion (Figure 1A). The level of HPV 16 infection in HNSCC group was significantly higher than the precarcinoma and normal oral mucous group (*P*<0.001), indicating that HPV 16 infection linked the malignant progression of oral mucous.

The examination results of the HPV16 expression and the demographic characteristics in the HNSCC cases were presented in Table 1. The data showed that HPV 16 positive cases tended to be younger and more likely to be never smokers and never drinkers compared with those without HPV 16 expression. The HPV 16 positive expression in oropharynx carcinoma was significantly higher than oral cancers (*P*=0.001). The HPV 16 positive expression in HNSCC patients associated with pathological grade of patients (*P*=0.034). However, there was no significant association of the HPV 16 status with sex and clinical stage of patients (*P*>0.05).

**Table 1:** HPV16 expression in peripheral blood and fresh tumor tissue samples of HNSCC in Southwest China and its association with clinicopathologic characteristics

Variable	HPV (PB)		*P*-value	HPV (TS)		*P*-value
	Negative	Positive		Negative	Positive	
**Age**			0.0101			0.0170
<60	60	29		60	25	
≥60	89	18		81	14	
**Gender**			0.897			0.804
Mae	84	27		80	23	
Female	65	20		61	16	
**Smoking status**			<0.001			0.0106
Current/former	86	10		74	11	
Never	63	37		67	28	
**Alcohol use**						0.0002
Positive	79	7	<0.001	77	8	
Negative	70	40		64	31	
**Clinical Stage**			0.422			0.978
I+II	63	23		69	19	
III+IV	86	24		72	20	
**Tumor Localization**			0.001			0.001
Mouth Floor	25	6		21	4	
Tongue	69	17		61	15	
Oropharyngeal	9	13		7	10	
Buccal Mucosa	46	11		52	10	
**Pathological Stage**			0.005			0.034
High	46	7		49	10	
Middle Grade	63	10		51	9	
Low Grade	40	30		41	20	

Chi-square was reported to compare the difference between HPV status and clinical-pathological characteristics. Former/current smokers defined as >= one pack-year history of smoking. Positive alcohol use was defined as current alcohol use of >one drink per day for 1 year (12 oz of beer with 5% alcohol, 5 oz of wine with 12% to 15% alcohol, or 1 oz of liquor with 45% to 60% alcohol).

### Concordance of HPV16 expression in different samples by different detection methods

The concordance of HPV16 by PCR, P16 immunostaining, and their combinations with measures of HPV16 was shown in Supplementary Table 1. The overall prevalence of HPV16 DNA in fresh tumor tissues by PCR was 21.67%, whereas the prevalence of HPV16 DNA in blood samples by PCR was 23.98%. The concordance between HPV16 DNA in fresh tumor tissues and blood samples was high with a k equivalent to 0.80(*P*=0.000). This data indicated the consistence of HPV expression in blood samples and tumor tissue. In paraffin sections, the overall prevalence of HPV16 DNA by PCR was 20.41% (40/196), and the prevalence of HPV16 by IHC was 26.53% (52/196). The concordance between PCR method and IHC in paraffin sections was high, suggesting that the HPV expression rate by PCR in paraffin sections was almost the same as the HPV expression detected by P16 IHC.

### MDSCs expression in peripheral blood and fresh tumor tissue samples of HNSCC in Southwest China

**Figure 2:**
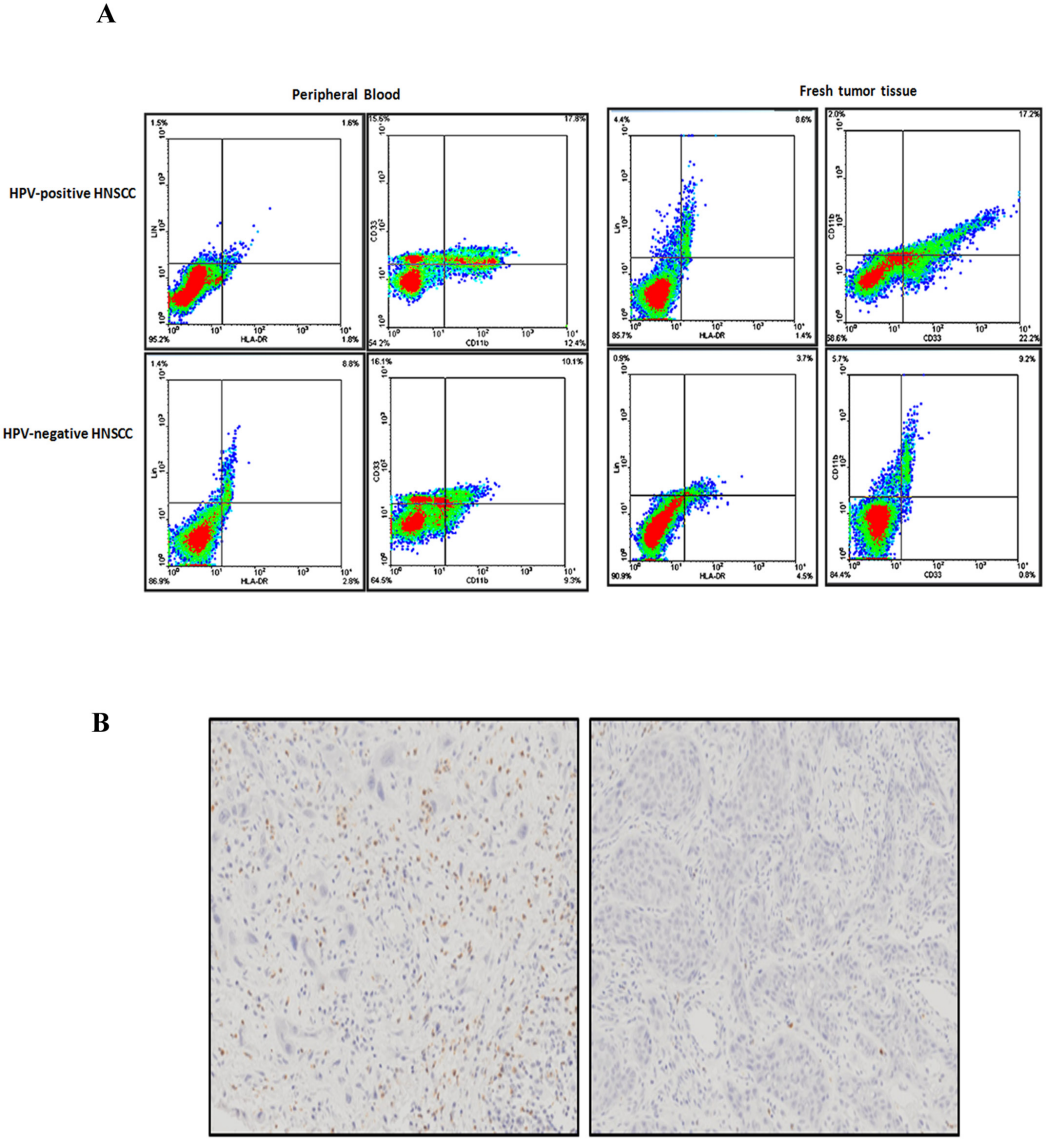
MDSCs expression in peripheral blood and fresh tumor tissue samples of HNSCC **A**. Representative flow cytometry image of CD11b+ LIN- HLA-DR- CD33+ MDSCs in peripheral blood and fresh tumor tissues of HPV-positive HNSCC and HPV-negative HNSCC. We first examined the percentage of LIN- HLA-DR- cells, and then screened the percentage of CD11b+ CD33+ cells in LIN- HLA-DR- cells. The peripheral blood sample and the fresh tumor tissue were from the same patients. The percentage of CD11b+ LIN- HLA-DR- CD33+ MDSCs in blood and tissue was almost the same. **B**. Representative immunohistochemical image of MPO in paraffin sections of HNSCC patients. Right was MPO in strong positive staining. Left was MPO in negative staining.

The balance between active immune responses against HPV and HPV-induced immune escape regulates viral clearance and carcinogenesis [23]. MDSCs are immature cells of myeloid origin, frequently found in tumor microenvironments and the blood of cancer patients, and contributed to tumor-mediated immune escape. However, the role and significance of MDSCs in HPV-associated HNSCC still remained blank. To elucidate the significance of MDSCs in HPV infection, we investigated the expression and level of MDSCs in HPV-related HNSCC. We applied the PE-Cy mouse anti-human CD11b, FITC-mouse anti-human LIN, APC mouse anti-human HLA-DR, PE mouse anti-human CD33 to examine the number of MDSCs in blood specimens of normal oral mucous, precancerous lesions, and HNSCC patients using flow cytometry. The results showed that the number of MDSCs was obviously up-regulated in the specimens of precancerous lesions (4.12%±0.96%) and HNSCC (10.22%±1.21%) in comparison with normal oral mucous(0.83%±1.25%, *P*=0.002). Then, to exclude the other disease resulting in the change of MDSCs in the peripheral blood of whole body, we detected the expression and level of MDSCs in fresh tumor tissue samples. The same results were obtained in the tissue samples of cases. Representative figure of the blood and tissue samples of one patient was presented in Figure 2A. In addition, we further used MPO, the marker of MDSCs in human clinic cases, to examine the expression of the paraffin specimens of MDSCs by immuohistochemistory. The positive brown staining of MPO was stained in the cytoplasm and nucleus of MDSC in light microscope (Figure 2B). The number of MDSCs was calculated and statistic result showed that MDSCs was higher expressed in HNSCC patients than normal oral mucous tissues. These data confirmed that MDSCs might be a critical cell population during the development of oral lesion to cancer.

Further, the analysis of the relationship between the expression of MDSCs and clinicopathologic features of HNSCC showed that the number of MDSCs in cases with T3-4 was more than cases with T1-2, and the number of MDSCs in cases with pathological grade II and III was more than that with grade I (*P=*0.002, *P=*0.001, respectively, Supplementary Table 2), however, there was no significant association of the MDSCs with age and gender of patients(*P*>0.05). This indicated that MDSCs may be recruited to HNSCC areas and the clinic stage and pathological grade of HNSCC patients may influence the recruitment of MDSCs.

### Relationship between HPV16 and MDSCs in HNSCC

We have found that the number of MDSCs were up-regulated in HNSCC. It was known that the evasion of host immunity employed by viruses to establish viral persistence has been shown to strikingly parallel mechanisms of tumor escape [24]. This prompted us to analyze the relationship between HPV16 infection and MDSCs number in HNSCC. We analyzed the expression of HPV16 and MDSCs in HNSCC and found that the number of MDSCs (13.84% ± 1.66%) in blood samples of 47 HPV-positive HNSCC was significantly higher than precancerous lesions and normal oral mucous (*P*=0.004). The same results were obtained in tumor tissue sample (Supplementary Table 3). This data indicated that HPV infection might promote MDSCs aggregation into HNSCC areas, which might constitute a concomitant microenvironment of viral and inflammation to plays an important role in HNSCC.

### LncRNAs microarry of HPV-related HNSCC

To further investigate the molecular mechanism of HPV16 accelerating MDSCs aggregation, we analyzed the lncRNAs profile of HPV-related HNSCC, which are emerging as a novel class of noncoding RNAs that are pervasively transcribed in the genome [25]. From the above HNSCC samples for HPV-positive HNSCC, HPV-negative HNSCC and normal oral mucous, we selected those characterized by RNA integrity preservation and high DNA quality (as assessed by spectrophotometric absorbance *A*260/*A*280 ratio and electrophoretic profile). These samples included 3 patients with the HPV-positive HNSCC, 3 patients with HPV-negative HNSCC, and 3 normal oral mucous classified as the controls. Feature Extraction software (version10.7.1.1, Agilent Technologies) was applied to process raw data and figures, and Genespring software (version 12.5; Agilent Technologies) was used to normalizate quantile of the raw data.

**Figure 3:**
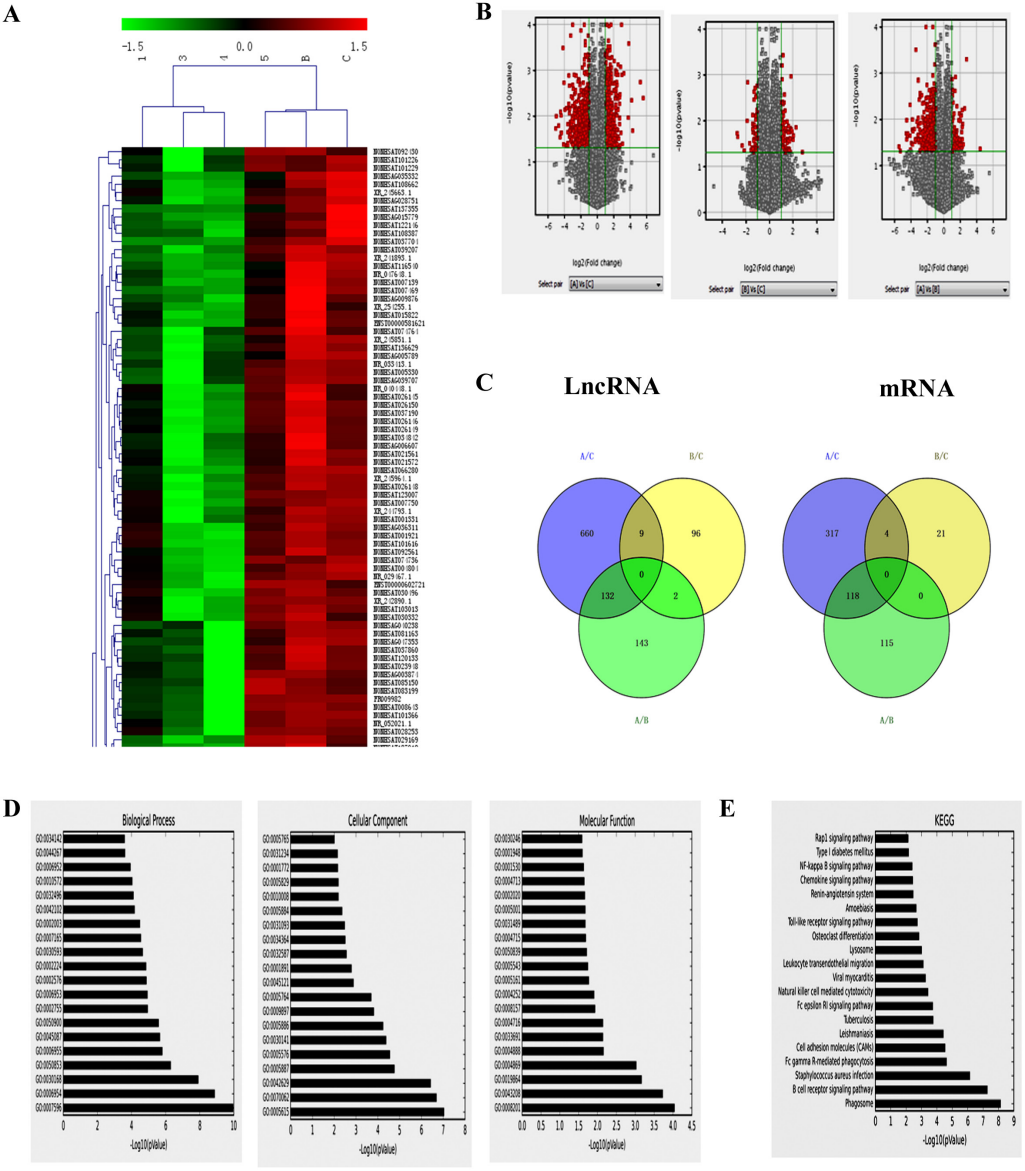
LncRNAs analysis of HPV-related HNSCC **A**. Hierarchical clustering analysis of differentially expressed lncRNAs between HPV-positive HNSCC and HPV-negative HNSCC. Red and green colors indicate high and low expression, respectively. In the heat map, columns represent samples and rows represent each lncRNA. **B**. Volcano plot of differentially expressed lncRNAs. The vertical lines correspond to 2.0-fold up and down and the horizontal line represents a *P* value of 0.05. A: HPV-positive group; B: HPV-negative group; C: normal oral mucous group. The gray spot showed fold change<2, *P*>0.05; and the red spot showed fold change≥2, P≤0.05. **C**. Venn analysis of the expressed lncRNAs among the control, HPV-positive HNSCC and HPV-negative HNSCC. **D**. The GO analytical data of aberrantly expressed mRNAs in biological process, cellular component and molecular function between HPV-positive HNSCC and HPV-negative HNSCC. **E**. The top 20 kinds of pathways among the differentially expressed transcripts between HPV-positive HNSCC and HPV-negative HNSCC by KEGG.

Differentially expressed lncRNAs and genes among the control, HPV-positive HNSCC and HPV-negative HNSCC were analyzed by unsupervised hierarchical clustering and the data were shown in the heat map of Figure 3A. We found that a total of 801 lncRNAs (396 up-regulated and 405 down-regulated) were significantly differently expressed in HPV-positive HNSCC compared with control group (fold change >=2), 107 lncRNAs (58 up-regulated and 49 down-regulated) were significantly differently expressed in HPV-negative HNSCC compared with control group (fold change > =2), and 277 lncRNAs (78 up-regulated and 199 down-regulated) were significantly differently expressed in HPV-positive HNSCC compared with HPV-negative HNSCC (fold change > = 2; Figure 3B). It was worth noting that there were 132 lncRNAs and 118 mRNAs in different HPV infected states of HNSCC (Figure 3C).

Twelve lncRNAs (5 up-regulated and 7 down-regulated) were significantly differently expressed in HPV-positive HNSCC compared with control group (fold change > = 10). There were 1 up-regulated and 10 down-regulated lncRNAs with more than 10-fold changes between HPV-positive HNSCC and HPV-negative HNSCC. Using the same criteria of lncRNAs, we found 28 up-regulated mRNAs, and 241 down-regulated mRNAs (fold change >= 2.0, *P* <0.05) between HPV-positive HNSCC and HPV-negative HNSCC. WFDC2 (fold change=44.73664) was the most down-regulated mRNA, and NONHSAT083749 (fold change=21.536058) was the most down-regulated lncRNA between HPV-positive HNSCC and HPV-negative HNSCC.

The GO analytical data of aberrantly expressed mRNAs between HPV-positive HNSCC and HPV-negative HNSCC (*P*< 0.05) showed that the most enriched GOs associated with differentially expressed transcripts were blood coagulation (GO: 000 7596; ontology: biological process; *P*=5.99e-10) “inflammatory response” (GO: 000 6954; ontology: biological process; *P*=3.28e-08) (Figure 3D Left), “extracellular space” (GO: 0005615; ontology: cellular component; *P*=2.43e-08) (Figure 3D Middle), and “heparin binding” (GO: 0008201; ontology: molecular function; *P*= 6.47e-04) (Figure 3D Right). Pathway analysis indicated that 187 kinds of pathways were significantly enriched among the differentially expressed transcripts between HPV-positive HNSCC and HPV-negative HNSCC (*P*< 0.05) (Figure 3E). The most enriched pathway was “Phagosome” (*P* = 5.03e-09), which was associated with 16 differentially expressed genes. Many of these pathways were linked to inflammation and immunity, such as “B cell receptor signaling pathway” (associated with 11 genes), “Natural killer cell mediated cytotoxicity” (associated with 9 genes), “Primary immunodeficiency” (associated with 3 genes), “Viral myocarditis” (associated with 6 genes).

### Construction of the co-expression network between mRNA and lncRNA in HPV-positive HNSCC

**Figure 4:**
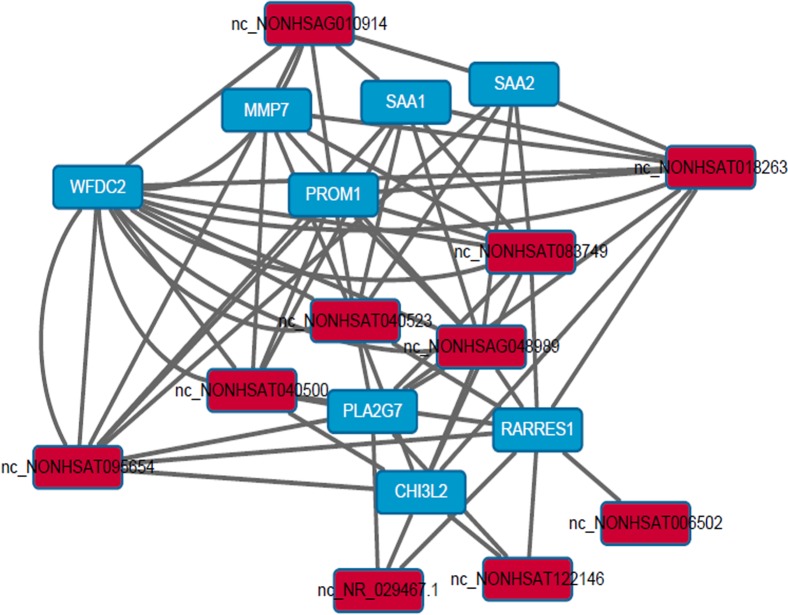
Construction of the co-expression network between mRNA and lncRNA in HPV-positive HNSCC The network of top 10 different mRNAs and top 10 different lncRNAs between HPV-positive HNSCC and HPV-negative HNSCC. The data showed that the top 10 different mRNAs closely associated with the top 10 different lncRNAs, and all the modules include down-regulated genes.

To explore the potential relationship between the 132 lncRNAs and 118 mRNAs, the correlated expression networks were done. The association between mRNA and lncRNA is close (pearson correlation, *P* <0.01), however, it is too complicated. Then, the network of top 10 different mRNAs and top 10 different lncRNAs between HPV-positive HNSCC and HPV-negative HNSCC was built and shown in Figure 4, which demonstrated that the top 10 different mRNAs closely associated with the top 10 different lncRNAs, and all the modules include down-regulated genes. In addition, the networks of top 10 different mRNAs and top 10 different lncRNAs between HPV-positive HNSCC and the control, and HPV-negative HNSCC and the control were built. These networks might indicate that lncRNAs regulated HPV+ HNSCC through nearby mRNAs.

### Validation of the microarray data using qRT-PCR

**Figure 5:**
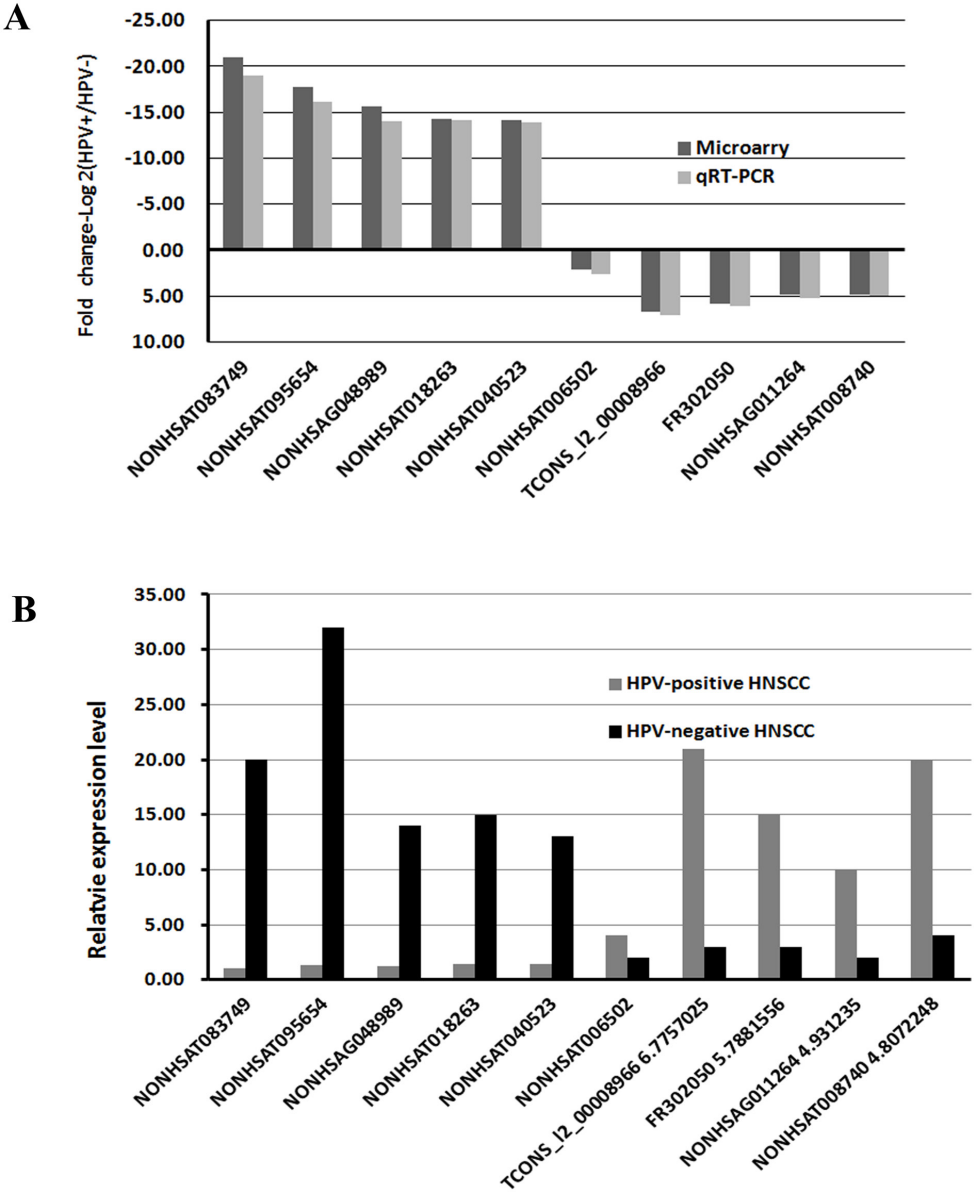
qRT-PCR validation of 10 differentially expressed lncRNAs **A**. Comparison of fold change of lncRNAs between HPV-positive and HPV-negative HNSCC. The data demonstrated the same trends of up-and down-regulation and the fold changes of qRT-PCR as the microarray data. **B**. The relative expression levels of top 10 lncRNAs in HPV-positive and HPV-negative HNSCC (*P* < 0.05). The result showed the same trends of regulation as the microarray data.

qRT-PCR was further performed to verify the lncRNA array results. We selected top 10 differentially expressed lncRNAs (NONHSAT083749, NONHSAT095654, NONHSAG048989, NONHS AT018263, NONHSAT0-40523, NONHSAT006502, TCONS_l2_00008966, FR302050, NONHSAG-011264, NONHSAT008740) between HPV-positive and HPV-negative group to detect their expression levels by qRT-PCR. The data demonstrated the same trends of up-and down-regulation and the fold changes of qRT-PCR as the microarray data (Figure 5A, 5B). Additionally, 10 differentially expressed mRNAs (WFDC2, SAA2, SAA1, MMP7, PROM1, RARRES1, CHI3L2, PLA2G7, CDKN2B, ZDHHC11) in the lncRNAs-mRNAs correlation networks were examined by qRT-PCR (Supplementary Figure 2). All the results showed the same trends of regulation as the microarray data. This validated that the results of qRT-PCR were in line with the microarray data.

### Expression of PROM1, CCAT1, HOTAIR, and MUC19 was associated with the recruitment of MDSCs in HPV-positive HNSCC

**Figure 6:**
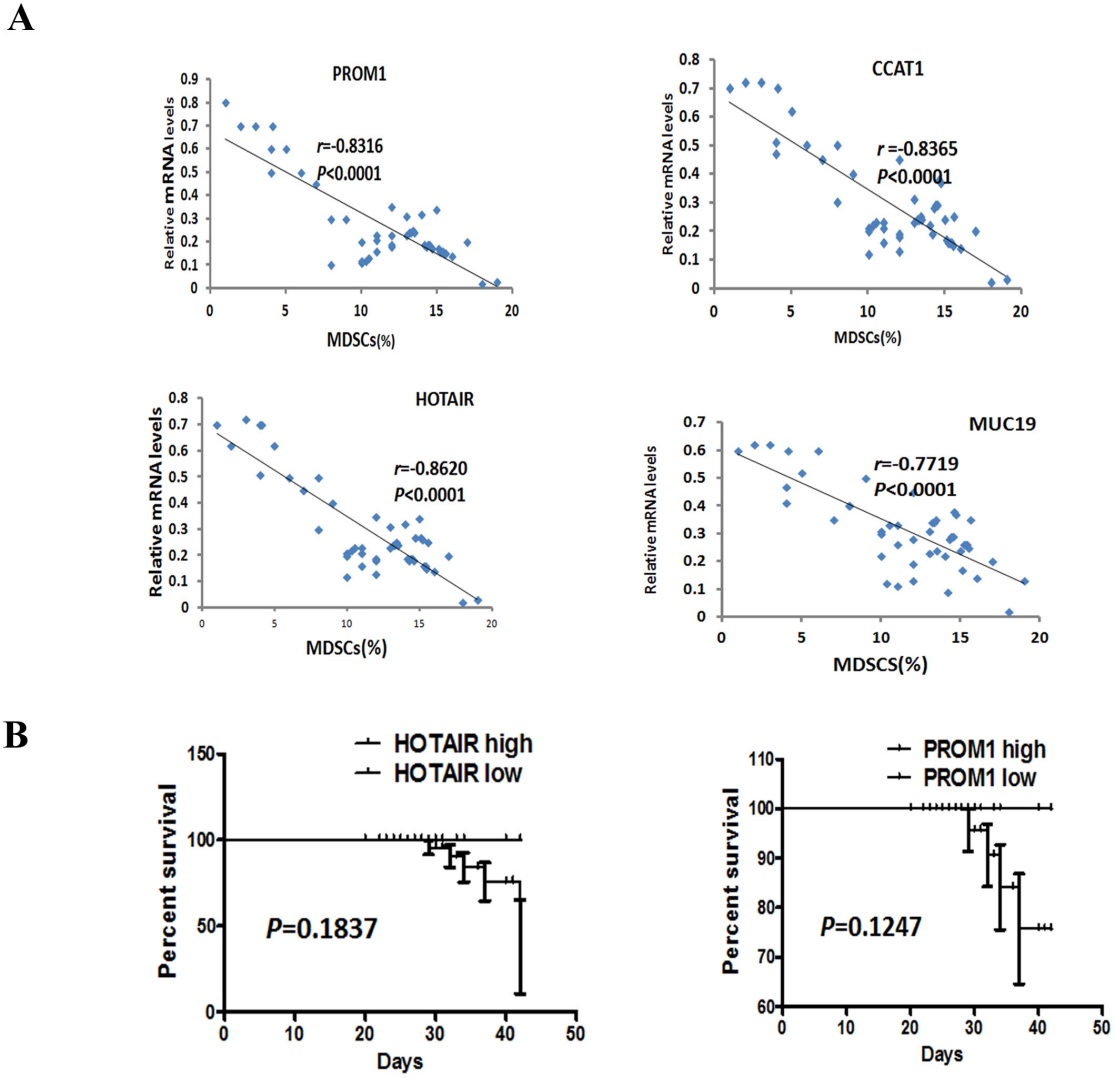
Correlation between the number of MDSCs and the transcript levels of HOTAIR, PROM1, CCAT1, and MUC19, and HOTAIR associated with the clinical outcome of HNSCC patients **A**. Correlation between the number of MDSCs and the transcript levels of HOTAIR, PROM1, CCAT1, and MUC19 in 47 HPV-positive HNSCC samples. The data showed that the level of HOTAIR, PROM1, CCAT1 and MUC19 were associated with the number of MDSCs. Statistical nonparametric comparison for correlation between the number of MDSCs and the transcript levels of lncRNAs was performed by Spearman method (r is indicated). **B**. Kaplan-Meier survival analysis in patients with HNSCC. Kaplan-Meier plot for OS and PFS with regard toHOTAIR and PROM1 in 47 HPV-positive HNSCC patients. *P* values were derived by log-rank/Mantel-Cox test. According to the relative value of RT-PCR, the cutoff of HOTAIR and PROM1 to differentiate high from low is 5, and more than 5 means high and others are low.

To investigate the role of lncRNAs in HPV16 promoting MDSCs aggregation in HNSCC, we examined 4 lncRNAs (HOTAIR, PROM1, CCAT1 and MUC19) expression in 47 HPV-positive blood samples using qRT-PCR. It turns out that the expression of HOTAIR, PROM1, CCAT1, and MUC19 was negatively associated with the number of MDSCs in HPV-positive HNSCC (Figure 6A).

**Figure 7:**
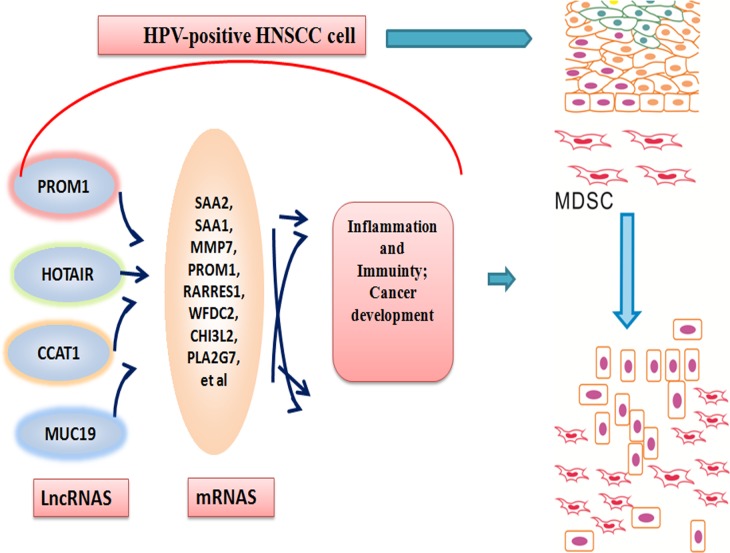
A model for the role of lncRNAs induced by HPV infection in the epithelial cells of oral mucous collecting MDSCs to progress to a tumor state Following transient HPV exposure, the epithelial cells of oral mucous activated a series of lncRNAs and their genes, which modified tumor inflammation and immunity microenvironment. This change contributed to the aggregations of MDSCs and cancer development.

To confirm the association of PROM1, HOTAIR, CCAT1, and MUC19 with the number of MDSCs in HPV-positive HNSCC, we examined their expressions for another independent HNSCC patient cohorts. The cohorts (Weifang), consisted of 30 patients of a current prospective study. qRT-PCR of HOTAIR, PROM1, CCAT1, and MUC19 in HNSCC patients revealed similar findings. The close correlation of HOTAIR, PROM1, CCAT1, and MUC19 with MDSCs in HPV-positive HNSCC has been shown in Figure 7.

Additionally, the survival curves were computed with the Kaplan-Meier method and compared between groups by using the log-rank test, though the following time is only 3-42 months. The result demonstrated that there was no significantly association between survive rate and the expression level of HOTAIR and PROM1 in 47 HPV-positive HNSCC (Figure 6B). The following-up will keep carrying on.

## DISCUSSION

The comparative genomic hybridization, whole exome sequencing and CpG island arrays have been applied to identify genomic imbalance, somatic gene mutations and CpG island hypermethylation in HPV-associated HNSCC [26–29]. These studies unraveled some difference in the genetic landscape and epigenetic alteration between HPV-negative and HPV-positive tumors. Recently, the role of lncRNAs in cancer has drawn great attention and lncRNAs are an enigmatic component of the whole transcriptome, which may involve in tumorigenesis, invasion and metastasis [30]. Here, we attempted to use lncRNA analysis to elucidate the difference of MDSCs recruitment between HPV-positive HNSCC and HPV-negative HNSCC, and discovered the role and significance of lncRNAs in HPV-associated HNSCC, indicating that lncRNA is a potential mediator of MDSCs recruitment in HPV-positive HNSCC.

HPV causes an epidemiologically and clinically distinct form of HNSCC [4, 31], yet the global prevalence of HPV in HNSCC remains unclear. Hammarstedt et al. [32] found that the distribution of HPV-positive tonsillar cancer cases was 23.3% in the 1970s, 29% in the 1980s, 57% in the 1990s and 68% during 2000-2002 in Stockholm, Sweden. Grulich group in Australia showed that ~35% of oropharyngeal cancers and ~25% of other oral cavity cancers are HPV positive and the incidence of HPV-related oral cavity and oropharyngeal cancers is increasing, whereas incidence at HPV-unrelated sites is decreasing [33]. OPSCC incidence increased from 1973 to 2004 in the United States, particularly among young individuals (< 60 years of age), men, and whites [34]. Kreimer et al. [35] found that the HPV positive rate of all HNSCC biopsy specimens was 25.9%, and 35.6% in oropharyngeal SCCs, 23.5% in oral SCCs and 24.0% in laryngeal SCCs through analyzing the data from 60 studies done in 26 countries. However, in China we still lack these relative data. This study is the first time to apply a large number of fresh tumor tissues and peripheral blood to examine HPV infection state in Southwest China. The distribution of HPV-positive HNSCC was shown in 23.98% in peripheral blood of HNSCC patients and 21.67% in tumor tissue. We observed strong agreement between tumor HPV status, as determined by PCR in peripheral blood and fresh tumor tissue. The positive rate of P16 in paraffin sections determined was almost the same as that the result of PCR for HPV 16 DNA. This demonstrated that P16 expression is a very good surrogate for tumor HPV status, though it is not specific for HPV type.

MDSCs have obtained much attention due to their roles in tumor immunity suppression as well as promotion of angiogenesis, invasion, and metastases. In our previous study, we showed that hypoxia could stimulate the migration of CD11b+Gr-1+ myeloid cells through increased production of macrophage migration inhibitory factor (MIF) and interleukin-6 (IL-6) in HNSCC cells [36]. The latest studies of MDSCs mainly focused on non-neoplastic infections, including bacterial, parasitic, fungal, and viral infections [24]. Here, in HNSCC patients we found that MDSCs levels were increased compared with normal oral mucous. The statistic analysis further confirmed that the number of MDSCs correlated positively the HPV infection state in HNSCC, indicating that HPV infection induced MDSCs accumulation and there was a link between HPV and MDSCs in HNSCC. This was in consistence with the previous reports [37]. It was found that there was significantly increased frequency of MDSCs observed in the spleens and peripheral blood of 4NQO-treated mice bearing oral cancer [38]. HPV16 E2 can promote anti-tumor innate effector function by modulating immunoregulatory events mediated by CD11b+Gr-1+ myeloid cells and their mediators [23].

To provide some molecular evidence for MDSCs collection in HPV-associated HNSCC, lncRNA analysis was applied to elucidate the difference between HPV-positive and HPV-negative HNSCC. We obtained 132 different lncRNAs in different HPV infected states of HNSCC, and confirmed the data by qRT-PCR in 2 much larger, independent HNSCC cohorts. This result was supported by previous findings. Gibb et al. [39] described the expression of 325 lncRNAs for the human oral mucosa, suggesting lncRNA expression contributes significantly to oral transcriptome. Detection of lncRNAs in saliva may be used as a noninvasive and rapid diagnostic tool for the diagnosis of oral cancer [40]. Then, we selected 4 genes for a more detailed analysis, based on their robust difference between HPV-positive and HPV-negative in larger HNSCC patients and their highly significant correlation between lncRNA and transcript levels in tumor samples. We found that HOTAIR, PROM1, CCAT1, and MUC19 not only characterized HPV-driven tumors, but also negatively correlated with MDSCs collection in HPV-positive HNSCC. These results were partly verified by previous data. CCAT1 in established human tumor cell lines or in tumor samples has been showed to play a critical role in the development, invasion and metastasis of colorectal cancer, and gastric cancer, mediate the EMT process, and be expected to be a new marker and treatment target in cancer diagnosis and treatment [41–44]. MUC19, PAICS, RBMXL1, KIF23 have been identified in melanoma, which might deserve further investigations the role in cancer [45–46]. HOTAIR could serve as one of the targets of the HPV16 oncoprotein E7 in the process of cervical carcinogenesis [47]. The role and significance of these lncRNAs in HPV-associated HNSCC will be deserved to investigate.

This work allows us to propose a model in which abnormally expressed lncRNAs occur at oral mucous following HPV infection and these lncRNAs may aggregate MDSCs and contribute to the progression of tumor formation (Figure 7). Cells infecting HPV 16 will change cell biologic consequences through three transforming proteins, E5, E6, and E7 [48]. The cell biologic change will first rely on lncRNA alteration, which will alter chromatin structure, regulate expression of protein-coding genes [49]. Here, MDSCs and lncRNA have been shown to be critical in modulating host immune mircoenvirnoment in HPV-positive HNSCC patients. Furthermore, the sensitivity and specificity of lncRNA biomarkers should be explored for the clinical evaluation of lncRNA as a molecular targeted approach to treat HPV-associated HNSCC patients.

## MATERIALS AND METHODS

### Patients and sample collection

One hundred and ninety-six patients with HNSCC who underwent resection of their tumors without preoperative chemotherapy, hormone therapy or radiotherapy at the Department of Oral and Maxillofacial Surgery, West China Hospital of Stomatology, Sichuan University between 2012 and 2015 were recruited for the study after giving informed consent. A complete medical history was obtained, and tumor assessment was performed at baseline (Table 1). The protocol of the study was approved by the Institutional Ethics Committee of the West China Medical Center, Sichuan University, China. In addition, 30 precancerous lesions and 30 normal oral mucous of healthy persons were included in this study.

After the completion of surgery, all the patients were reviewed every 3 months during the first 2 years, every 6 months during the subsequent 3 to 5 years, and once per year thereafter until death or data censoring. OS (overall survival) was counted from the date of random assignment to the date of death. PFS (progression-free survival) were counted from the date of random assignment to loco-regional recurrence, distant metastasis, or death resulting from any cause. The average follow-up time of all of the patients was 20 months (range 3-42 months) till August 10, 2015 and it will continue to follow-up.

### HPV infection status

Fresh tissues were cut in half for parallel RNA and genomic DNA extraction with the TRIzol reagent (Invitrogen) and the QIAamp Mini Kit (Qiagen), respectively. For PCR amplification of the HPV 16, 100 ng of DNA was amplified using consensus primers HPV 16 (457-bp product). PCR primer sequence for HPV 16 is Forward 5- CAC AGT TAT GCA CAG AGC TGC-3; Reverse 5-CATATATTCATGCAATGTAGGTGTA-3.

### Immuohistochemistory (IHC)

IHC of P16 and MPO, human MDSC marker, was performed on 4-mm-cut representative sections of paraffin-embedded samples by the streptavidin-peroxidase method followed.

### MDSCs examination by flow cytometry

Cells were analyzed using Flow Cytometer (Cytomic FC500, Beckman). Tissues were trim into 1-2mm^3^ tissue block and put into burnisher. Then cell suspension was collected and centrifugated. To blood specimen, heparin sodium and PBS/Hanks was added into blood of individuals. Ficol was used to separate lymphocyte. Serum free medium containing 1% BSA was added into cell suspension, and then incubated on ice for 10 minutes. PE-Cy Mouse Anti-Human CD11b, FITC-Mouse Anti-Human LIN, APC Mouse Anti-Human HLA-DR, PE Mouse Anti-Human CD33 (BD Biosciences) were added to cell suspension and incubated on ice for 30 minutes. Cells were washed and resuspended in 500 mL buffer and analyzed.

### Microarray hybridization and bioinformatic analysis

Total RNAs were extracted using Trizol reagent (Invitrogen, Carlsbad, CA, USA) following manufacturer's instruction. Microarray hybridization and bioinformatic analysis were done according to the manufacturer's instruction. Feature Extraction software (version10.7.1.1, Agilent Technologies) was applied to process raw data and figures, and Genespring software (version 12.5; Agilent Technologies) was used to normalizate quantile of the raw data.

### Validation by quantitative real time polymerase chain reaction (qRT-PCR)

qRT-PCR analysis was performed for selected lncRNAs. The total RNA extraction with Trizol reagent (Invitrogen, Carlsbad, CA, USA) and cDNA preparation (High capacity cDNA reverse transcription kit, life technology, USA) were done following manusfactures’ instruction. The total RNA of specimens was collected with TRIzol reagent (Invitrogen) and treated with RNase-free DNase I (Takara) to avoid genomic DNA contamination. The complementary (c)DNA was stored at −80°C until used for PCR. PCR amplification of the cDNA template was done using Thunderbird SYBR qPCR mix (TOYOBO) on ABI PRISM 7300 sequence detection system (Applied Biosystems).

### Statistical analysis

The difference of MDSCs number between HPV-positive and HPV-negative groups was evaluated with *t*-test. Chi-square was used to analyze the associations between HPV status and clinical-pathological characteristics. Correlation between the number of MDSCs and the transcript levels of lncRNAs was performed by Spearman method. The Kaplan–Meier method was applied for survival analysis and the statistical significances between the groups were evaluated using the log-rank/Mantel-Cox test. All the statistical analyses were performed using SPSS 13.0 (SPSS Inc., Chicago, IL, USA). Statistical analysis was considered to be significant when the probability value <0.05.

## SUPPLEMENTARY FIGURES AND TABLES



## References

[1] Leemans CR, Braakhuis BJ, Brakenhoff RH The molecular biology of head and neck cancer. Nat Rev Cancer.

[2] Rothenberg SM, Ellisen LW The molecular pathogenesis of head and neck squamous cell carcinoma. J Clin Invest.

[3] Lewin F, Norell SE, Johansson H, Gustavsson P, Wennerberg J, Biörklund A, Rutqvist LE Smoking tobacco, oral snuff, and alcohol in the etiology of squamous cell carcinoma of the head and neck: a population-based case-referent study in Sweden. Cancer.

[4] D'Souza G, Kreimer AR, Viscidi R, Pawlita M, Fakhry C, Koch WM, Westra WH, Gillison ML Case-control study of human papillomavirus and oropharyngeal cancer. N Engl J Med.

[5] Fakhry C, Gillison ML Clinical implications of human papillomavirus in head and neck cancers. J Clin Oncol.

[6] Li G, Sturgis EM The role of human papillomavirus in squamous carcinoma of the head and neck. Curr Oncol Rep.

[7] Ringström E, Peters E, Hasegawa M, Posner M, Liu M, Kelsey KT Humanpapillomavirus type 16 and squamous cell carcinoma of the head and neck. Clin Cancer Res.

[8] Senft E, Lemound J, Stucki-Koch A, Gellrich NC, Kreipe H, Hussein K Expression of cyclin-dependent kinase inhibitor 2A 16, tumour protein 53 and epidermal growth factor receptor in salivary gland carcinomas is not associated with oncogenic virus infection. Int J Oral Sci.

[9] Rischin D, Young RJ, Fisher R, Fox SB, Le QT, Peters LJ, Solomon B, Choi J, O'Sullivan B, Kenny LM, McArthur GA Prognostic significance of p16INK4A and human papillomavirus in patients with oropharyngeal cancer treated on TROG 02.02 phase III trial. J Clin Oncol.

[10] Fakhry C, Westra WH, Li S, Cmelak A, Ridge JA, Pinto H, Forastiere A, Gillison L Improved survival of patients with human papillomavirus-positive head and neck squamous cell carcinoma in a prospective clinical trial. J Natl Cancer Inst.

[11] Gillison ML, D'Souza G, Westra W, Sugar E, Xiao W, Begum S, Viscidi R Distinct risk factor profiles for human papillomavirus type 16-positive and human papillomavirus type 16-negative head and neck cancers. J Natl Cancer Inst.

[12] Fatica A, Bozzoni I Long non-coding RNAs: new players in cell differentiation and development. Nat Rev Genet.

[13] Kogo R, Shimamura T, Mimori K, Kawahara K, Imoto S, Sudo T, Tanaka F, Shibata K, Suzuki A, Komune S, Miyano S, Mori M Long noncoding RNA HOTAIR regulates polycomb-dependent chromatin modification and is associated with poor prognosis in colorectal cancers. Cancer Res.

[14] Wang F, Ren S, Chen R, Lu J, Shi X, Zhu Y, Zhang W, Jing T, Zhang C, Shen J, Xu C, Wang H, Wang H Development and prospective multicenter valuation of the long noncoding RNA MALAT-1 as a diagnostic urinary biomarker or prostate cancer. Oncotarget.

[15] Li T, Xie J, Shen C, Cheng D, Shi Y, Wu Z, Deng X, Chen H, Shen B, Peng C, Li H, Zhan Q, Zhu Z Upregulation of long noncoding RNA ZEB1-AS1 promotes tumor metastasis and predicts poor prognosis in hepatocellular carcinoma. Oncogene.

[16] Wang C, Yan G, Zhang Y, Jia X, Bu P Long non-coding RNA MEG3 suppresses migration and invasion of thyroid carcinoma by targeting of Rac1. Neoplasma.

[17] Gao W, Chan JY, Wong TS Long non-coding RNA deregulation in tongue squamous cell carcinoma. Biomed Res Int.

[18] Liu B, Sun L, Liu Q, Gong C, Yao Y, Lv X, Lin L, Yao H, Su F, Li D, Zeng M, Song E A cytoplasmic NF-κB interacting long noncoding RNA blocks IκB hosphorylation and suppresses breast cancer metastasis. Cancer Cell.

[19] Zhou Q, Huang XR, Yu J, Yu X, Lan HY (2015). Long noncoding RNA Arid2-IR Is a novel herapeutic target for renal inflammation. Mol Ther.

[20] Liu S, Song L, Yao H, Zhang L, Xu D, Gao F, Li Q MiR-375 is epigenetically downregulated by HPV-16 E6 mediated DNMT1 upregulation and modulates EMT of cervical cancer cells by suppressing lncRNA MALAT1. PLoS One.

[21] Srikantan V, Zou Z, Petrovics G, Xu L, Augustus M, Davis L, Livezey JR, Connell T, Sesterhenn IA, Yoshino K, Buzard GS, Mostofi FK, McLeod DG PCGEM1, a prostate-specific gene, is overexpressed in prostate cancer. Proc Natl Acad Sci U S A.

[22] Gabrilovich DI, Nagaraj S Myeloid-derived suppressor cells as regulators of the immune system. Nat Rev Immunol.

[23] Sunthamala N, Pientong C, Ohno T, Zhang C, Bhingare A, Kondo Y, Azuma M, Ekalaksananan T HPV16 E2 protein promotes innate immunity by modulating immunosuppressive status. Biochem Biophys Res Commun.

[24] Goh C, Narayanan S, Hahn YS Myeloid-derived suppressor cells: the dark knight or the joker in viral infections?. Immunol Rev.

[25] ENCODE Project Consortium An integrated encyclopedia of DNA elements in the human genome. Nature.

[26] Dahlgren L, Mellin H, Wangsa D, Heselmeyer-Haddad K, Björnestål L, Lindholm J, Munck-Wikland E, Auer G, Ried T, Dalianis T Comparative genomic hybridization analysis of tonsillar cancer reveals a different pattern of genomic imbalances in human papillomavirus-positive and –negative tumors. Int J Cancer.

[27] Smeets SJ, Braakhuis BJ, Abbas S, Snijders PJ, Ylstra B, van de Wiel MA, Meijer GA, Leemans CR, Brakenhoff RH Genome-wide DNA copy number alterations in head and neck squamous cell carcinomas with or without oncogene-expressing human papillomavirus. Oncogene.

[28] Agrawal N, Frederick MJ, Pickering CR, Bettegowda C, Chang K, Li RJ, Fakhry C, Xie TX, Zhang J, Wang J, Zhang N, El-Naggar AK, Jasser SA Exome sequencing of head and neck squamous cell carcinoma reveals inactivating mutations in NOTCH1. Science.

[29] Kostareli E, Holzinger D, Bogatyrova O, Hielscher T, Wichmann G, Keck M, Lahrmann B, Grabe N, Flechtenmacher C, Schmidt CR, Seiwert T, Dyckhoff G, Dietz A HPV-related methylation signature predicts survival in oropharyngeal squamous cell carcinomas. J Clin Invest.

[30] Gutschner T, Diederichs S The hallmarks of cancer: a long non-coding RNA point of view. RNA Biol.

[31] Schwartz SM, Daling JR, Doody DR, Wipf GC, Carter JJ, Madeleine MM, Mao EJ, Fitzgibbons ED, Huang S, Beckmann AM, McDougall JK, Galloway DA Oral cancer risk in relation to sexual history and evidence of human papillomavirus infection. J Natl Cancer Inst.

[32] Hammarstedt L, Lindquist D, Dahlstrand H, Romanitan M, Dahlgren LO, Joneberg J, Creson N, Lindholm J, Ye W, Dalianis T, Munck-Wikland E Human papillomavirus as a risk factor for the increase in incidence of tonsillar cancer. Int J Cancer.

[33] Grulich AE, Jin F, Conway EL, Stein AN, Hocking J Cancers attributable to human papillomavirus infection. Sex Health.

[34] Chaturvedi AK, Engels EA, Anderson WF, Gillison ML Incidence trends for human papillomavirus-related and –unrelated oral squamous cell carcinomas in the United States. J Clin Oncol.

[35] Kreimer AR, Clifford GM, Boyle P, Franceschi S Human papillomavirus types in head and neck squamous cell carcinomas worldwide: a systematic review. Cancer Epidemiol Biomarkers Prev.

[36] Zhu G, Tang Y, Geng N, Zheng M, Jiang J, Li L, Li K, Lei Z, Chen W, Fan Y, Ma X, Li L, Wang X, Liang X HIF-α/MIF and NF-κB/IL-6 axes contribute to the recruitment of CD11b+Gr-1+ myeloid cells in hypoxic microenvironment of HNSCC. Neoplasia.

[37] Ostrand-Rosenberg S Immune surveillance: a balance between protumor and antitumor immunity. Curr Opin Genet Dev.

[38] Chu M, Su YX, Wang L, Zhang TH, Liang YJ, Liang LZ, Liao GQ Myeloid-derived suppressor cells contribute to oral cancer progression in 4NQO-treated mice. Oral Dis.

[39] Gibb EA, Enfield KS, Stewart GL, Lonergan KM, Chari R, Ng RT, Zhang L, MacAulay CE, Rosin MP, Lam WL Long non-coding RNAs are expressed in oral mucosa and altered in oral premalignant lesions. Oral Oncol.

[40] Tang H, Wu Z, Zhang J, Su B Salivary lncRNA as a potential marker for oral squamous cell carcinoma diagnosis. Mol Med Rep.

[41] Ye Z, Zhou M, Tian B, Wu B, Li J Expression of lncRNA-CCAT1, E-cadherin and N-cadherin in colorectal cancer and its clinical significance. Int J Clin Exp Med.

[42] Deng L, Yang SB, Xu FF, Zhang JH Long noncoding RNA CCAT1 promotes hepatocellular carcinoma progression by functioning as let-7 sponge. J Exp Clin Cancer Res.

[43] Ma MZ, Chu BF, Zhang Y, Weng MZ, Qin YY, Gong W, Quan ZW Long non-coding RNA CCAT1 promotes gallbladder cancer development via negative modulation of miRNA-218-5p. Cell Death Dis.

[44] Yang F, Xue X, Bi J, Zheng L, Zhi K, Gu Y, Fang G Long noncoding RNA CCAT1, which could be activated by c-Myc, promotes the progression of gastric carcinoma. J Cancer Res Clin Oncol.

[45] Cifola I, Pietrelli A, Consolandi C, Severgnini M, Mangano E, Russo V, De Bellis G, Battaglia C Comprehensive genomic characterization of cutaneous malignant melanoma cell lines derived from metastatic lesions by whole-exome sequencing and SNP array profiling. PLoS One.

[46] Young HW, Williams OW, Chandra D, Bellinghausen LK, Pérez G, Suárez A, Tuvim MJ, Roy MG, Alexander SN, Moghaddam SJ, Adachi R, Blackburn MR, Dickey BF, Evans CM Central role of Muc5ac expression in mucous metaplasia and its regulation by conserved 5′ elements. Am J Respir Cell Mol Biol.

[47] Sharma S, Mandal P, Sadhukhan T, Roy Chowdhury R, Ranjan Mondal N, Chakravarty B, Chatterjee T, Roy S, Sengupta S Bridging links between long Noncoding RNA HOTAIR and HPV oncoprotein E7 in cervical cancer pathogenesis. Sci Rep.

[48] Hellner K, Münger K Human papillomaviruses as therapeutic targets in human cancer. J Clin Oncol.

[49] Braconi C, Kogure T, Valeri N, Huang N, Nuovo G, Costinean S, Negrini M, Miotto E, Croce CM, Patel T microRNA-29 can regulate expression of the long non-coding RNA gene MEG3 in hepatocellular cancer. Oncogene.

