# Supplementary material for: LncRNAs as an intermediate in HPV16 promoting myeloid-derived suppressor cell recruitment of head and neck squamous cell carcinoma
Source: Oncotarget. 2017 Feb 1;8(26):42061–75. doi: 10.18632/oncotarget.14939 (PMC5522049; doi:10.18632/oncotarget.14939)
Supplement: Supplementary file 1 [file oncotarget-08-42061-s001.pdf]

LncRNAs as an intermediate in HPV16 promoting myeloid-derived suppressor cell recruitment of head and neck squamous cell carcinoma

SUPPLEMENTARY MATERIALS

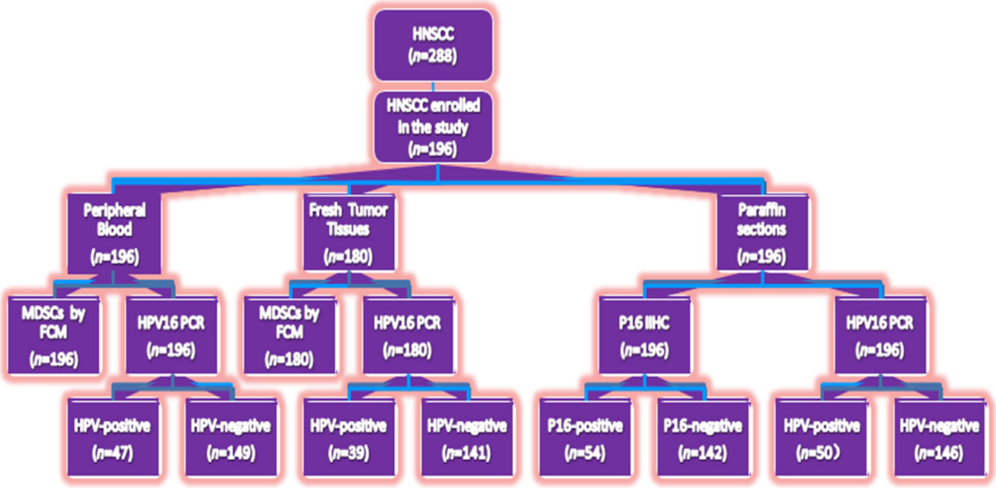

Supplementary Figure 1: Schematic diagram of this study.

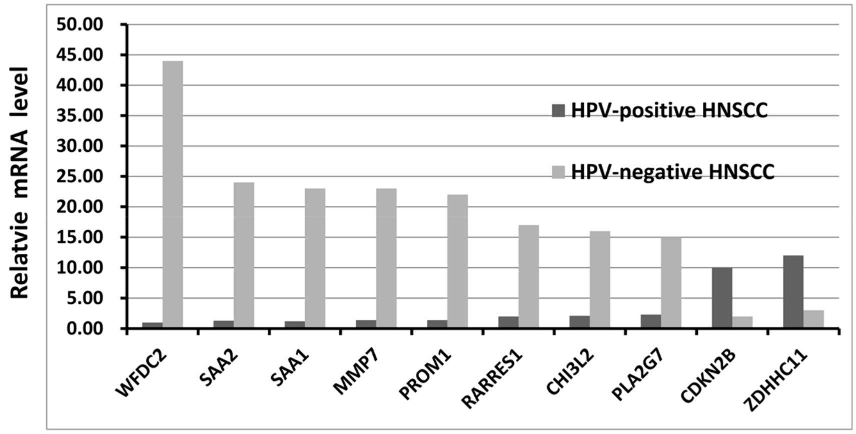

Supplementary Figure 2: qRT-PCR validation of 10 differentially expressed mRNAs. The relative expression levels of top 10 mRNAs in HPV-positive and HPV-negative HNSCC($P < 0.05$). The result showed the same trends of regulation as the microarray data.

Supplementary Table 1: The concordance of HPV16 by PCR and P16 IHC in peripheral blood and tumor tissues

HPV Markers	Status	HPV 16 DNA (fresh tumor tissues) by PCR			*P* value
		Positive(*n*%)	Negative(*n*%)	Kappa	
HPV 16 DNA (peripheral blood) by PCR				0.80	0.000
	Positive	34(87.2)	11(7.8)		
	Negative	5(12.8)	130(92.2)		
HPV 16 DNA (paraffin section) by PCR				0.88	0.000
	Positive	30(76.9)	10(7.1)		
	Negative	9(23.1)	131(92.9)		
P 16 (paraffin section) by IHC				0.66	0.040
	Positive	34(87.2)	18(12.8)		
	Negative	5(12.8)	123(87.2)		

Supplementary Table 2: The expression of MPO in HNSCC patients by ICH and its association with clinicopathologic characteristics

Variable	*n*	MPO		*P*-value
		Negative	Positive	
**Age**				0.566
<60	89	43	46	
≥60	107	57	50	
**Gender**				0.150
Male	111	62	49	
Female	85	38	47	
**Clinical stage**				0.002
T1-T2	86	68	18	
T3-T4	110	32	78	
**Pathological stage**				0.001
Grade I	53	28	15	
Grade II+ III	143	62	81	

Supplementary Table 3: The number of MDSCs in HNSCC patients, precancerous lesions and normal oral mucous tissues by FCM

Group	*n*	MDSCs in peripheral blood(%)	*P* value	*n*	MDSCs in tumor tissue(%)	*P* value
HPV-positive HNSCC	47	13.84 ± 1.66	0.004	39	15.78± 2.64	<0.001
Precancerous lesions	30	4.12±0.96		30	5.12±1.47	
Normal oral mucous tissues	30	0.83±1.25		30	0.67±2.14	
